# Complete genome sequence of *Clostridium perfringens* CBA7123 isolated from a faecal sample from Korea

**DOI:** 10.1186/s13099-017-0181-1

**Published:** 2017-06-02

**Authors:** Yeon Bee Kim, Joon Yong Kim, Hye Seon Song, Changsu Lee, Joseph Kwon, Jisu Kang, Jin-Kyu Rhee, Myeong Seon Jeong, Young-Do Nam, Seong Woon Roh

**Affiliations:** 1 Microbiology and Functionality Research Group, World Institute of Kimchi, Gwangju, 61755 Republic of Korea; 2 Biological Disaster Analysis Group, Korea Basic Science Institute, Daejeon, 34133 Republic of Korea; 3 Gut Microbiome Research Group, Korea Food Research Institute, Seongnam, 13539 Republic of Korea; 4 University of Science and Technology, Daejeon, 34113 Republic of Korea; 5 Department of Food Science and Engineering, Ewha Womans University, Seoul, 03760 Republic of Korea; 6 Chuncheon Center, Korea Basic Science Institute, Gangneung, Gangwon-do 24341 Republic of Korea

**Keywords:** *Clostridium perfringens*, Complete genome sequence, Comparative genomic analysis, Pathogenesis, Antimicrobial resistant, Virulence factor

## Abstract

**Background:**

*Clostridium perfringens* is an opportunistic human pathogen that causes necrotic enteritis, mild diarrhea, clostridial myonecrosis or gas gangrene, sepsis, etc. In this study, we aim to determine the pathogenesis of this bacterium at the genomic level. The genome of strain CBA7123 was sequenced, and a comparative genomic analysis between strain CBA7123 and four other related *C. perfringens* strains was performed.

**Results:**

The genome of strain CBA7123 consisted of one circular chromosome and one plasmid that were 3,088,370 and 46,640 bp long with 28.5 and 27.1 mol% G+C content, respectively. The genomic DNA was predicted to contain 2798 open reading frames (ORFs), 10 rRNA genes, and 94 tRNA genes. The genomic comparison analysis between the five strains revealed the distinctive virulence properties of strain CBA7123 by highlighting certain strain-specific genes.

**Conclusions:**

In this study, the *C. perfringens* CBA7123 genome was sequenced and compared with other *C. perfringens* genomes. Among the various genes sequenced, the detection of antimicrobial resistance genes and those encoding various virulence factors may extend the understanding of the pathogenesis of *C. perfringens* strains.

**Electronic supplementary material:**

The online version of this article (doi:10.1186/s13099-017-0181-1) contains supplementary material, which is available to authorized users.

## Background

*Clostridium perfringens* is a Gram-positive, spore-forming, strict anaerobic, rod-shaped bacterium belonging to the phylum *Firmicutes* [1–3]. This bacterium inhabits diverse environments such as soil, sewage, and animal intestines [4]. Although *C. perfringens* does not invade healthy cells, it acts as a pathogen by producing various enzymes and toxins and is also considered a common cause of food poisoning worldwide [5]. *C. perfringens* toxins, mainly comprising α, β, ε, and ι extracellular toxins, are indicators for classifying its strains as A to E toxinotypes [6–8]. The type A of *C. perfringens* is the most commonly found toxinotype that possesses only α toxin-encoding gene, *plc*. Strains with α-toxin genes can cause necrotic enteritis, mild diarrhea, clostridial myonecrosis or gas gangrene, sepsis, and food poisoning in humans as well as various enterotoxemic diseases in livestock [7, 9, 10]. In this study, we aim to elucidate the pathogenesis of this species through its genome, specifically the virulence-related genes. The genome of *C. perfringens* CBA7123 was completely sequenced and analyzed using bioinformatics. Additionally, genomic data that were compared between strain CBA7123 and four other *C. perfringens* strains would illustrate the virulence mechanisms of these bacteria.

## Methods

### Isolation and DNA extraction of *C. perfringens* CBA7123

*Clostridium perfringens* CBA7123 (=KCCM 43242) was isolated from the feces of a 73-year-old man, and a pure culture was obtained using serial dilution. The colony was cultured anaerobically in ATCC medium no. 2840, modified Eggerth–Gagnon medium (10 g peptone, 4 g Na_2_HPO_4_·2H_2_O, 2 g porcine gastric mucin, 50 ml sheep blood, and 15 g agar per liter) at 37 °C for 24 h. The total genomic DNA of strain CBA7123 was extracted using QuickGene DNA tissue kit S (Kurabo, Japan) and the G-spin total DNA extraction kit (iNtRON Biotechnology, Korea). The purity, quality, and quantity of genomic DNA were measured using Agilent 2100 Bioanalyzer (Agilent Technologies, USA) as described the manufacturer’s instruction.

### Library preparation and genome sequencing

Detailed genome sequencing was performed as described previously [11]. Briefly, the genomic DNA of strain CBA7123 was sheared according to the PacBio 20-kb Template Preparation using BluePippin Size-Selection System protocol, and SMRTbell library was prepared using P6-C4 chemistry (Pacific Biosciences, USA). The sequences were obtained using PacBio RS II system (Pacific Biosciences) as following an instruction of the manufacturer. The 150,292 reads were generated with 7090 bp of average read length by the PacBio RS II system from one SMRT cell.

### Genome assembly and annotation

De novo assembly of the genome sequence was performed using Hierarchical Genome Assembly Process (HGAP) version 2 software, with default parameters supported by PacBio SMRT Analysis ver. 2.3.0 [12]. rRNA and tRNA of the assembled sequence were identified using RNAmmer 1.2 and tRNAscan-SE 1.21, respectively. Genes were predicted using Glimmer3 of Rapid Annotation using Subsystem Technology (RAST) server (http://rast.nmpdr.org), and functional gene annotations were performed using the SEED, Clusters of Orthologous Groups (COG, http://www.ncbi.nlm.nih.gov/COG), and Kyoto Encyclopedia of Genes and Genomes (KEGG, http://www.genome.jp/kegg/) databases. PathogenFinder 1.1 [13] and ResFinder 2.1 [14] were used to estimate the pathogenicity and antimicrobial resistance genes, respectively. The virulence factors were searched using the Basic Local Alignment Search Tool (BLAST) in the virulence factors of pathogenic bacteria database [15] with default parameters and predicted with zero e-value.

### Comparative genomic analysis

The *C. perfringens* strains for comparative genomic analysis were selected using Microbial Nucleotide BLAST in the NCBI complete genome database. The four strains with BLAST total scores over 5.5e+06 were selected: strains FORC 003, JP55, FORC 025, and JP838. To compare the genomic structures between strain CBA7123 and these four strains, the progressive alignment algorithm in MAUVE multiple genome alignment software 2.4.0 was used [16]. The OrthoANI algorithm was used to analyze genomic relatedness between strain CBA7123 and other *C. perfringens* species. OrthoANI percentages were calculated and a phylogenetic tree was constructed, as described by Lee et al. [17]. Orthologs between strain CBA7123 and the reference strains were predicted and mapped using the reciprocal best hit method in UBLAST [18]. Pan-genome orthologous groups (POGs) were estimated using the EzBioCloud Comparative Genomics Database (http://cg.ezbiocloud.net/) [19], and their presence was calculated using the Jaccard coefficient. UPGMA clustering was then used to show clustering between strain CBA7123 and the reference strains as a dendrogram, based on the presence or absence of gene content. The intersections and differences of POG sets of the different strains were visualized as a Venn diagram, using the jvenn program [20].

### Quality assurance

The single colony of strain CBA7123 was transferred more than three times to obtain a pure culture and confirmed using a variable pressure field emission scanning electron microscope (Chuncheon Center, Korea Basic Science Institute, Korea) (Additional file 1: Figure S1) and a PCR-sequencing approach targeting 16S rRNA gene as described by Min et al. [21].

## Results and discussion

### General features

The genome of strain CBA7123 comprised two circular contigs that were 3,088,370 and 46,640 bp long with 28.5 and 27.1 mol% G+C content, respectively. Each contig matched to a chromosome and plasmid by the BLAST analysis with GenBank genome database. 2798 open reading frames (ORFs), 10 rRNA (10 5S rRNA, 10 16S rRNA, 10 23S rRNA) operons, and 94 tRNA genes were present. Among the various COG categories, G (carbohydrate transport and metabolism; 205 ORFs), E (amino acid transport and metabolism; 168 ORFs), J (translation, ribosomal structure, and biogenesis; 163 ORFs), K (transcription; 149 ORFs), and L (replication, recombination, and repair; 147 ORFs) made up the largest proportion (≥6% of the total COGs classifications). Detailed genome features are summarized in Table 1, and numbers of COG functional categories are shown in Table 2.

**Table 1:** General features of the *Clostridium perfringens* CBA7123 genome

Property	Term
Finishing quality	Complete
Libraries used	SMRTbell library
Sequencing platforms	PacBio_20K
Assemblers	PacBio SMRT analysis 2.3.0
Pre-filtered reads	150,292
Post-filtered reads	77,985
Average genome coverage	282.57X
Genome size (bp)	3,135,010
DNA G+C content	28.5
Total ORFs	2798
rRNA operons	10
tRNA genes	94

**Table 2:** Number of general COG-associated functional genes

Code	Value	% age	Description
J	163	6.65	Translation, ribosomal structure and biogenesis
K	149	6.08	Transcription
L	147	6.00	Replication, recombination and repair
D	27	1.10	Cell cycle control, cell division, chromosome partitioning
O	71	2.90	Posttranslational modification, protein turnover, chaperones
M	139	5.67	Cell wall/membrane/envelope biogenesis
N	4	0.16	Cell motility
P	125	2.10	Inorganic ion transport and metabolism
T	96	3.92	Signal transduction mechanisms
C	137	5.59	Energy production and conversion
G	205	8.37	Carbohydrate transport and metabolism
E	168	6.86	Amino acid transport and metabolism
F	77	3.14	Nucleotide transport and metabolism
H	83	3.39	Coenzyme transport and metabolism
I	54	2.20	Lipid transport and metabolism
Q	18	0.73	Secondary metabolites biosynthesis, transport and catabolism
R	0	0.00	General function prediction only
S	787	32.12	Function unknown
Total	2450	100.00	

### Comparative genomic analysis of strain CBA7123 with other *C. perfringens* strains

**Fig. 1:**
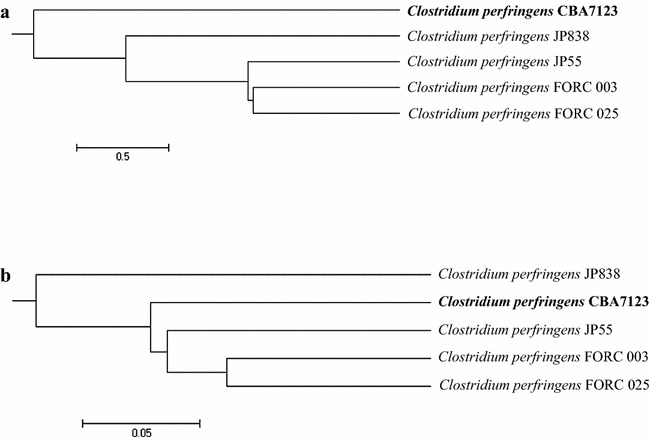
Phylogenetic tree depicting the relationship between *Clostridium perfringens* CBA7123 and four reference strains: FORC 003, FORC 025, JP55, and JP838, constructed based on **a** OrthoANI values using the orthologous average nucleotide identity tool and **b** gene content (presence or absence) using Jaccard coefficients and UPGMA clustering

**Fig. 2:**
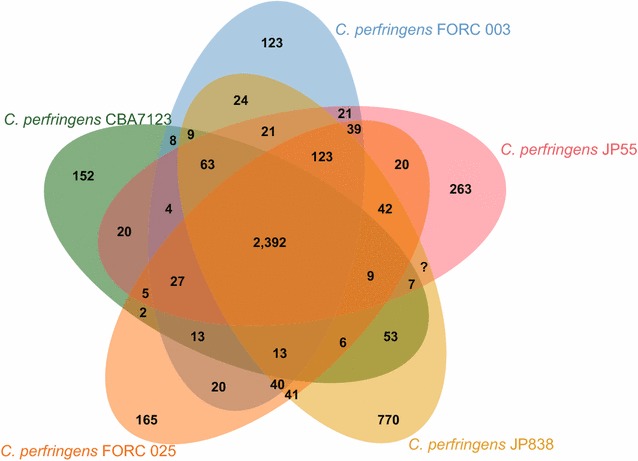
Venn diagram based on POGs indicating the orthologous groups among five *Clostridium perfringens* strains. Number of Venn diagrams represents number of shared genes among the genomes of the five strains

In the comparison of genomic structures between strain CBA7123 and strains FORC 003, JP55, FORC 025, and JP838, locally collinear blocks indicated high homology (Additional file 1: Figure S2). OrthoANI values between strain CBA7123 and each of strains FORC 003, JP55, FORC 025, and JP838 were 96.2, 96.2, 96.3, and 95.8, respectively. Although the phylogenetic tree constructed based on these OrthoANI values did not obviously indicate that strain CBA7123 was clustered with the other *C. perfringens* strains (Fig. 1a), all of the OrthoANI values were over 95%, which is the cut-off for species demarcation [17]. A POG comparison showed that the five strains shared 2392 POGs; however, 152 POGs were exclusive to strain CBA7123 (Fig. 2). Of the 152 POGs, some POGs encoded for antibiotic resistance and antiviral defense functions: two kinds of multidrug export MepA proteins, CRISPR-associated endonuclease Cas1 and Cas2, 5′ to 3′ exodeoxyribonuclease. The dendrogram created based on the presence of POGs indicated that strain CBA7123 was clustered with strains JP55, FORC 003, and FORC 025 (Fig. 1b). These results revealed that strain CBA7123 is closely related to *C. perfringens*, but different from other *C. perfringens* strains.

### Pathogenesis, virulence factors, and antimicrobial resistance genes

Strain CBA7123 was determined to be a human pathogen with 0.77 probability, using PathogenFinder 1.1. The 13 proteins except for conserved hypothetical proteins and hypothetical proteins were matched with pathogenic organisms belonging to class *Clostridia* (Table 3). The genome contained tetracycline-resistance genes: two *tetA*(P) genes, one each in the chromosome and plasmid; and one *tetB*(P) gene in the plasmid. Strain FORC 003 genome also contained the same tetracycline-resistance genes, whereas strain JP838 genome only contained *tetA*(P). No antimicrobial resistance genes were detected in the other two strains. The key virulence factors of strain CBA7123 were predicted to be exo-alpha-sialidase (*nanI*), sialidase (*nanH*), collagenase (*colA*, kappa-toxin), perfringolysin O (*pfoA*, theta-toxin), phospholipase C (*plc*, alpha-toxin), and alpha-clostripain (*cloSI*) with 96, 95, 98, 98 and 96% identity, respectively, in the chromosome and all of which were also present in the four reference strains. Sialidase hydrolyzes the α-linkage of terminal sialic acid residues from mammalian cell surface to generate free sialic acid, which is important carbon and energy source for colonization and growth of bacterial pathogen in the intestines [22]. Perfringolysin O and phospholipase C are soluble toxin and phospholipid cleaving enzyme leading to host cell lysis, respectively, and these two enzymes have synergistic effects in *Clostridium perfringens*-mediated gas gangrene [23, 24]. Collagenase and alpha-clostripain are not major determinant of virulence in clostridial myonecrosis, but it could be assumed that the two proteins have effects on *C. perfringens*-mediated disease [25, 26]. Strain CBA7123 could be classified as toxinotype A, based on the presence of the α-toxin gene (*plc*). Identification of tetracycline-resistance genes and various other virulence factors may help future research into reducing the pathogenesis of *C. perfringens* in humans and contribute to faster treatment response.

**Table 3:** Pathogenesis-related proteins matched to the genus *Clostridium*

Matched protein function	Matched organisms	Protein ID
Acetyltransferase GNAT family	*Clostridium perfringens* ATCC 13124	ABG84563
Conserved hypothetical protein	*Clostridium perfringens* str. 13	BAB81124
Creatininase	*Clostridium perfringens* ATCC 13124	ABS84729
DedA family protein	*Clostridium perfringens* ATCC 13124	ABG84228
FemAB family protein	*Clostridium perfringens* SM101	ABG87847
Iron-sulfur cluster-binding protein	*Clostridium perfringens* SM101	ABG85421
Oxidoreductase, FAD-binding	*Clostridium perfringens* SM101	ABG85949
PTS system, mannose/fructose/sorbose family, IIC component	*Clostridium perfringens* ATCC 13124	ABG82202
Putative csfB protein	*Clostridium perfringens* SM101	ABG87739
Putative membrane protein	*Clostridium perfringens* ATCC 13124	ABG83481
Transcriptional regulator, PadR family	*Clostridium perfringens* ATCC 13124	ABG82435
Transcriptional regulator, PadR family	*Clostridium perfringens* SM101	ABG85373
TrkA domain protein	*Clostridium perfringens* ATCC 13124	ABG84483

### Future directions

The genome information of strain CBA7123 can improve the understanding of *C. perfringens*, and the information of its antimicrobial resistance and virulence factors can contribute to the development of methods for preventing *C. perfringens*-related food poisoning. Future research studies should investigate the pathogenesis mechanism in detail as well as the specific roles of each virulence factor.

## Additional file

**Additional file 1: Figure S1.** A photomicrograph of *Clostridium perfringens* strain CBA7123 using Variable Pressure Field Emission Scanning Electron Microscope (VP-FE-SEM). **Figure S2.** Comparison of genomic structure between *Clostridium perfringens* CBA7123 and strains FORC 003, FORC 025, JP55, and JP838, using a progressive alignment algorithm in Mauve. The locally collinear blocks with identical colors represent highly homologous regions. The genomes were figured based on scale of the genome of strain CBA7123.

## Abbreviations

BLAST: Basic Local Alignment Search Tool
COGs: Clusters of Orthologous Groups
HGAP: Hierarchical Genome Assembly Process
KEGG: Kyoto Encyclopedia of Genes and Genomes
ORFs: open reading frames
POGs: pan-genome orthologous groups
RAST: Rapid Annotation using Subsystem Technology
