# Supplementary material for: Complete genome sequence of Clostridium perfringens CBA7123 isolated from a faecal sample from Korea
Source: Gut Pathog. 2017 Jun 2;9:32. doi: 10.1186/s13099-017-0181-1 (PMC5457660; doi:10.1186/s13099-017-0181-1)

Additional Files

Complete genome sequence of *Clostridium perfringens* CBA7123 isolated from Korean feces

Yeon Bee Kim[1], Joon Yong Kim[1], Hye Seon Song[1], Changsu Lee[1], Joseph Kwon[2], Jisu Kang[3,4], Jin-Kyu Rhee[5], Myeong Seon Jeong[6], Young-Do Nam[3,4†] and Seong Woon Roh[1†*]

[1]Microbiology and Functionality Research Group, World Institute of Kimchi, Gwangju 61755, Republic of Korea

[2]Biological Disaster Analysis Group, Korea Basic Science Institute, Daejeon 34133, Republic of Korea

[3]Gut Microbiome Research Group, Korea Food Research Institute, Seongnam 13539, Republic of Korea

[4]University of Science and Technology, Daejeon 34113, Republic of Korea

[5]Department of Food Science and Engineering, Ewha Womans University, Seoul 03760, Republic of Korea

[6]Chuncheon Center, Korea Basic Science Institute, Gangwon-do 24341, Republic of Korea

† Young-Do Nam and Seong Woon Roh contributed equally to this work

*Corresponding author:

S.W. Roh: Microbiology and Functionality Research Group, World Institute of Kimchi, Gwangju 61755, Republic of Korea. Tel: +82 62 610 1778; Fax: +82 62 610 1853; E-mail: swroh@wikim.re.kr

Additional File 1: Fig. S1. A photomicrograph of *Clostridium perfringens* strain CBA7123 using Variable Pressure Field Emission Scanning Electron Microscope (VP-FE-SEM).

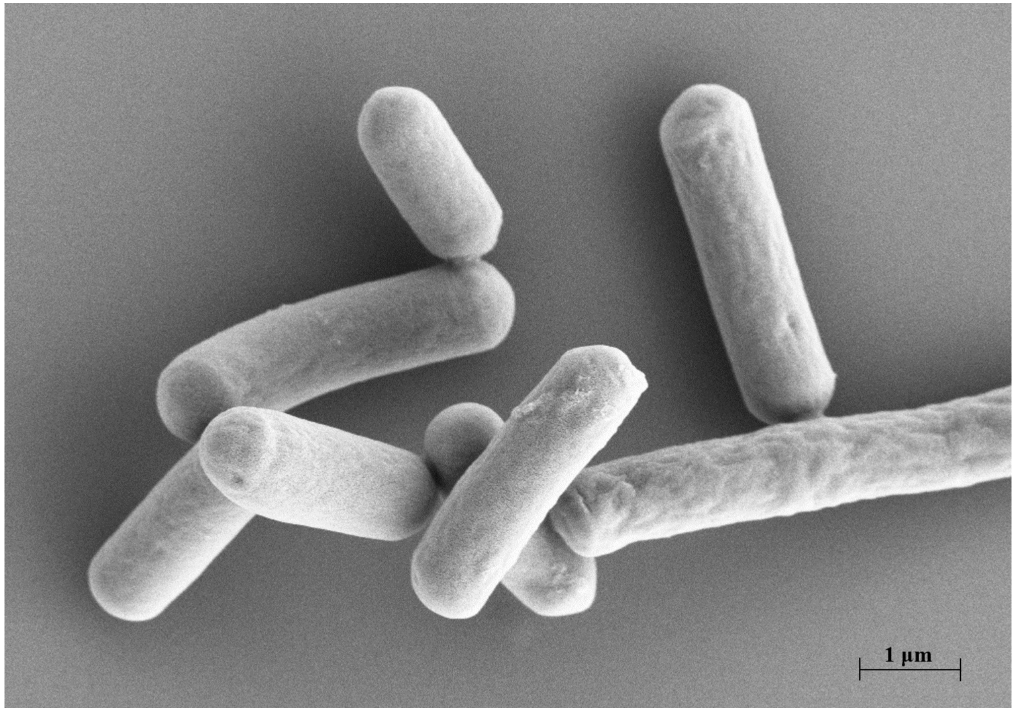

Additional File 1: Fig. S2. Comparison of genomic structure between *Clostridium perfringens* CBA7123 and strains FORC 003, FORC 025, JP55, and JP838, using a progressive alignment algorithm in Mauve. The locally collinear blocks (LCBs) with identical colors represent highly homologous regions. The genomes were figured based on scale of the genome of strain CBA7123.

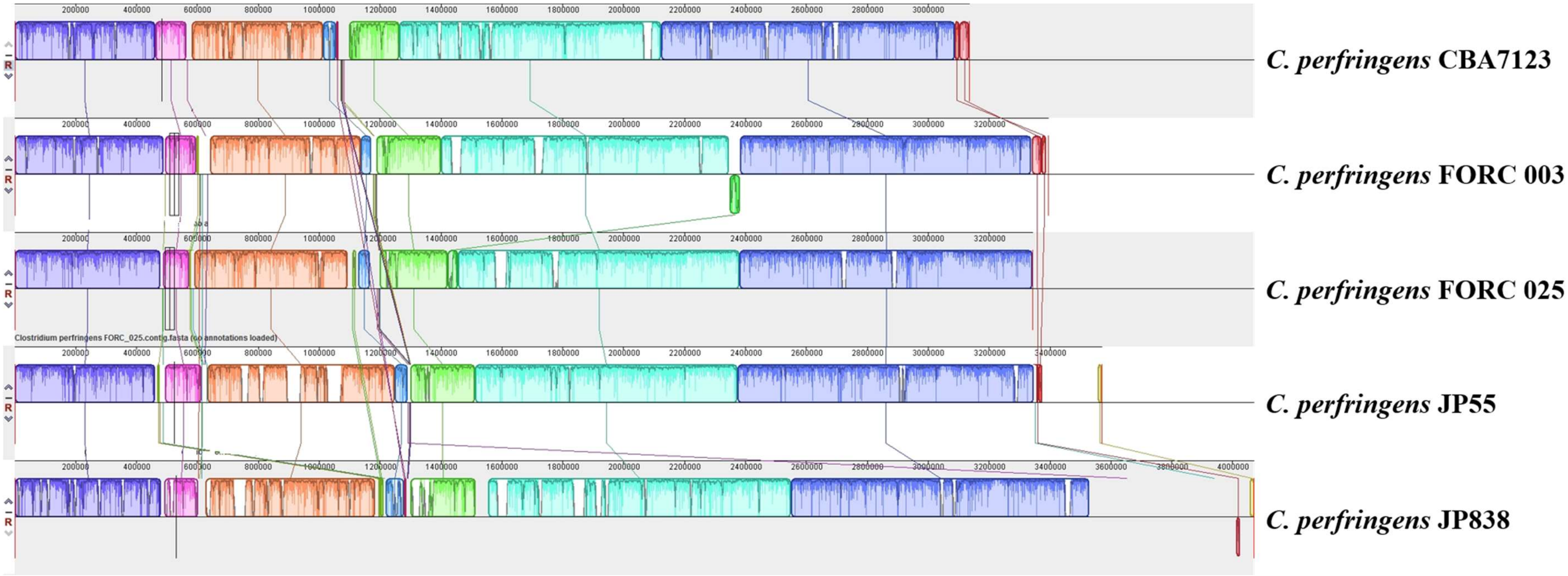

Supplement: Supplementary file 1 — Additional file 1: Figure S1. A photomicrograph of Clostridium perfringens strain CBA7123 using Variable Pressure Field Emission Scanning Electron Microscope (VP-FE-SEM). Figure S2. Comparison of genomic structure between Clostridium perfringens CBA7123 and strains FORC 003, FORC 025, JP55, and JP838, using a progressive alignment algorithm in Mauve. The locally collinear blocks with identical colors represent highly homologous regions. The genomes were figured based on scale of the genome of strain CBA7123. [file 13099_2017_181_MOESM1_ESM.pdf]
